# A Longitudinal Study of the Effects of Ketogenic Diet on Seizures, Cardiorespiration, Sleep Architecture and Mortality in the Kv1.1 Knockout Mouse Model of Sudden Unexpected Death in Epilepsy (SUDEP)

**DOI:** 10.3390/nu18050809

**Published:** 2026-03-01

**Authors:** Shruthi H. Iyer, Stephanie A. Matthews, Jodi Hallgren, Lauren Netzel, Timothy A. Simeone, Kristina A. Simeone

**Affiliations:** Department of Pharmacology & Neuroscience, Creighton University School of Medicine, Omaha, NE 68178, USAstephaniematthews@creighton.edu (S.A.M.); jodihallgren@creighton.edu (J.H.);

**Keywords:** epilepsy, sudden unexpected death, *Kcna1*-null, heart rate, respiration, apnea, hypoxemia, ketogenic diet, metabolic therapy, SUDEP biomarkers, longevity

## Abstract

**Background**: Sudden unexpected death in epilepsy (SUDEP) causes significant mortality, affecting approximately 1 in 1000 people with epilepsy. Clinical and preclinical studies have identified severe seizures, bradycardia, apnea, severe postictal hypoxia, and sleep deficiency that emerge prior to SUDEP and thus may represent temporal biomarkers. The metabolic ketogenic diet (KD) therapy increases longevity in preclinical SUDEP models. Here, the hypothesis that KD therapy would determine whether the emergent sleep deficiency, bradycardia, apnea and/or hypoxemia persist as temporal biomarkers in preclinical SUDEP was tested. **Methods**: Kv1.1 knockout (KO) mice, a preclinical SUDEP model, and wild-type littermates were weaned onto a standard diet (SD) or treated with KD. In separate cohorts, approximately every 10 days, seizures and sleep architecture were recorded with electroencephalography–electromyography (EEG-EMG), heart rate was measured with noninvasive ECGenie, apnea was assessed with noninvasive airway mechanics, and blood O_2_ saturation was measured with pulse oximetry. Data were aligned from the day of sudden death and analyzed retrospectively. **Results**: KD treatment significantly increased longevity and reduced seizures, reproducing previous studies. Using retrospective analyses from the day of death, KD treatment attenuated the emergence of (i) interictal intermittent bradycardia in the last 20 days of life, (ii) apnea, and (iii) intermittent hypoxemia in the last 10 days of life. In contrast, (iv) KD treatment did not rescue REM and NREM sleep deficiencies during the last 10 days of life. **Conclusions**: Our findings provide novel preclinical support for KD as a candidate therapy to attenuate seizure frequency and burden, bradycardia, apnea, and hypoxemia in SUDEP. In addition, sleep deficiency persisted as a potential temporal biomarker of preclinical SUDEP; however, causality will need to be tested in future studies.

## 1. Introduction

Approximately 1 in 1000 people with epilepsy (PWE) succumb to SUDEP, making it a leading cause of mortality in epilepsy [1,2,3]. The conceptual notion that SUDEP risk is dynamic and can be lowered depends in part on the occurrence of ‘comorbid modifiable conditions’ that can be targeted to personalize therapeutic strategies for postponing and preventing SUDEP [4,5]. However, a comprehensive understanding of risk factors and whether they are modifiable is lacking; thus, pathophysiology impacting risk is a topic of current clinical and preclinical research.

There is a small number of factors and genetic mutations that are associated with increased SUDEP risk. It is clear that risk increases to 1 in 150 for individuals with refractory seizures, constituting ~30% of PWE. Severe refractory generalized tonic–clonic (GTC) seizures, cardiac arrhythmias, changes in heart rate variability, postictal central apnea and postictal hypoxemia are physiological conditions that are associated with increased SUDEP risk; antiseizure medication noncompliance also increases risk [6,7,8,9,10,11]. In addition, mutations in multiple genes associated with epilepsy and SUDEP have been identified, including in the *Kcna1* gene (which encodes the alpha subunit of the Kv1.1 potassium channel) [12,13,14,15]. However, experiencing risk factors or having genetic mutations of increased risk does not dictate whether a PWE will experience SUDEP.

Use of preclinical models allows for the discovery and verification of risk factors (perhaps modifiable) and treatments to delay or prevent SUDEP. Kv1.1 knockout (KO) mice have spontaneous recurrent seizures and model temporal lobe epilepsy, and 100% of mice experience sudden death prematurely, thus providing utility as a SUDEP model [16,17,18]. KO mice have a cardiorespiratory pathophysiology that resembles clinical risk factors, consisting of intermittent interictal apnea, hypoxemia and bradyarrhythmia [4,16,19,20,21]. An additional risk factor discovered in high-SUDEP-risk KO mice is a deficiency in rapid eye movement (REM) and non-rapid eye movement (NREM) sleep [22]. These preclinical findings have been subsequently supported by clinical evidence of sleep disorder comorbidities in a notable percentage of cases in the North American SUDEP Registry [10]. In addition, a recent case–control clinical study reported that nuanced changes in slow-wave sleep power are associated with greater SUDEP risk [23].

An important nuance of these preclinical risk factors is that they are absent in younger low-SUDEP-risk cohorts and present in the older high-SUDEP-risk cohorts, suggesting they are temporal in nature [4,19,22]. Indeed, lifelong actimetry monitoring has revealed that the rest deficiency *emerges* in the last 10–15 days of life (note: the term ‘rest’ is used in actimetry due to the lack of electroencephalography-based identification of REM sleep and NREM sleep) [24]. The emergence of a risk factor prior to death is supported by anecdotal evidence from a survey of caregivers who recalled sleep problems in SUDEP victims in the weeks prior to death [5]. In addition to the emergence of sleep problems, a clinical case report described bradyarrhythmia prior to SUDEP when compared to recordings seven months earlier [25].

Herein, we will use the term ‘temporal biomarker’ to indicate a change in physiology that emerges prior to sudden death. Whether the emergent physiological changes that arise prior to death are temporal biomarkers that indicate proximity to sudden death or mechanistically contribute to sudden death is unknown. To begin to address this knowledge gap, we hypothesized that temporal biomarkers would persist in cohorts administered a treatment that delays but does not prevent sudden death. The treatment selected to delay sudden death in KO mice was the ketogenic diet (KD) [18]. The KD is a metabolic therapy that has been used in epilepsy management for over a century [26]. Clinical studies demonstrate that the KD can reduce seizure frequency by more than 50% in approximately two-thirds of individuals with refractory seizures, a known SUDEP risk factor, and confer seizure freedom in up to 13% of patients [27,28]. In addition, KD therapy has been shown to exert beneficial effects on sleep, cardiac function, apnea, and blood oxygen saturation in clinical and preclinical studies [24,29,30,31,32]. However, whether KD can mitigate these temporal biomarkers prior to sudden death in epilepsy is unknown.

Here, we tested the hypothesis that KD therapy would determine whether the emergent sleep deficiency, bradycardia, apnea and/or hypoxemia persist as temporal biomarkers in preclinical SUDEP.

## 2. Materials and Methods

### 2.1. Animals

Congenic *Kcna1* heterozygous mice on the C3HeB/FeJ background strain were bred in cages with nestlets and reared in a small and quiet room in the Animal Resource Facility at Creighton University to minimize stress. The room was temperature- and humidity-controlled and maintained on a 12 h light/dark cycle. Weaned *Kcna1*-null (referred to as Kv1.1 knockout (KO)) mice and wild-type (WT) littermates were provided access to food and water ad libitum. The genotype of the mice was determined from tail clips by Transnetyx, Inc. (Cordova, TN, USA). On post-natal day (P) 21, KO and WT groups were randomly weaned onto either a standard diet (SD; 2018S Teklad Global Rodent Diets, 44% carbohydrate, 18% protein, 6.2% fat, Inotiv, Madison, WI, USA) or KD (3.2% carbohydrate, 8.6% protein, 75% fat, with a ratio of 6.3:1 fat/carbohydrate/protein; Bio-Serv F3666, Frenchtown, NJ, USA). Mice remained on the respective diet until KO cohorts died of natural mortality, at which point the paired WT littermate was euthanized at the same age. An equal number of male and female mice were represented in each group. To increase rigor and to avoid a potential metabolic confounding variable, KD cohorts were kept in cages with specialized paper bedding (7099P TEK-Fresh Laboratory Animal Bedding; Inotiv, Inc., West Lafayette, IN, USA); SD cohorts were kept on standard corn cob bedding (7092–7097 Teklad Corncob Bedding; Inotiv). Animal care, monitoring, and procedures were in accordance with National Institutes of Health Guidelines and the United States Public Health Service’s Policy on Humane Care and Use of Laboratory Animals. Experimental protocols were approved by the Institutional Animal Care and Use Committee at Creighton University School of Medicine. Estimated sample sizes were determined with previous data a priori via power analysis. If statistical significance was achieved with a smaller sample size, additional subjects were not included in accordance with these policies. SUDEP Common Data Elements were used in the design of this study to increase rigor, reproducibility, and translational relevance of (i) core and death-related information, (ii) neurological variables, (iii) physiologic measures, and (iv) therapeutics and pharmacology [33,34]. Experimental designs were in accordance with the ARRIVE 2.0 guidelines.

### 2.2. Experimental Design

**Figure 1:**
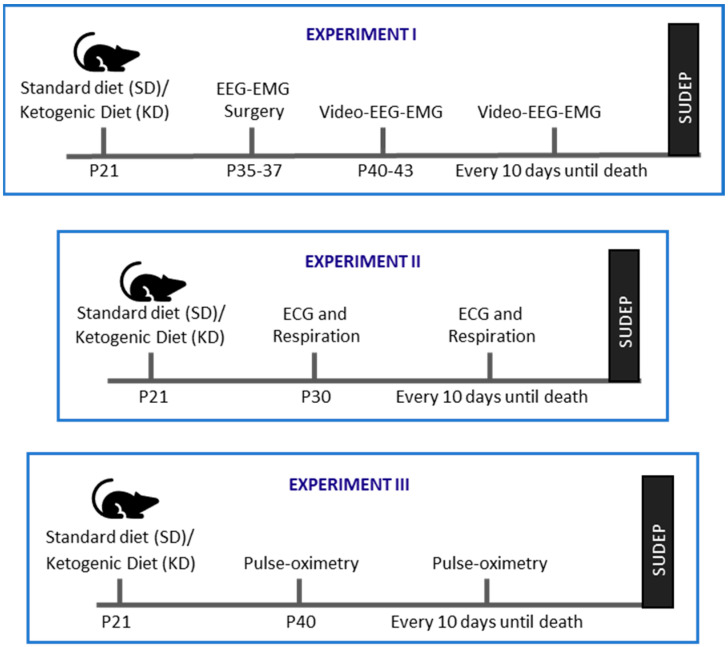
Design schematics for Experiments I–III. A different cohort of KO mice and WT littermates was used for each experiment.

Three experimental cohorts were used in this study (Figure 1). The *n* for each sample size reflects a single animal. To minimize circadian influence, experiments were conducted between zeitgeber time (ZT) 03:00 and 09:00 h (ZT 00:00 h is lights on). Experiments were powered to assess differences in endpoints. If sex differences were not observed with 3–4 males and females, then sex was not included as a variable. If sex differences were noted, then the power analyses were recalculated to include sex as a variable. After exclusion criteria were applied to the data sets, this study used a total of 72 subjects. In the experiments below, when a KO mouse experienced sudden death, their paired WT littermate was euthanized on the same day.

Experiment I: In the first cohort of animals, mice were subjected to electroencephalography–electromyography (EEG-EMG) surgery on ~P35–37. Seizures and sleep architecture were recorded for 48 h. approximately every 10 days until sudden death in KO mice.

Experiment II: In the second cohort, starting on P30, electrocardiography (ECG) and respiratory endpoints, and behavioral seizures were recorded approximately every 10 days throughout their lifespan until sudden death.

Experiment III: In the third cohort, blood oxygen saturation was determined via pulse oximetry approximately every 10 days starting at ~P40 until sudden death in KO mice.

### 2.3. Video-Electroencephalography (EEG) and Electromyography (EMG)

Surgeries were conducted using aseptic techniques. KO and WT mice (~P35–37) were anesthetized with 3–5% isoflurane, and EEG and EMG electrodes were implanted. Two subdural, ipsilateral cortical electrodes were implanted at 1.2 mm anterior to bregma and 1 mm lateral to midline, and another at 1.5 mm posterior to bregma and 1 mm lateral to midline. A ground electrode was implanted 1.5 mm posterior to the bregma and 1 mm lateral to the midline. EMG electrode wires were inserted into the nuchal muscles. Electrodes were soldered to the head mount (Pinnacle Technologies, Inc., Lawrence, KS, USA) and secured to the skull with Loctite superglue. The surrounding skin was treated with Betadine, local anesthesia (1–3 drops of 0.25% lidocaine), and an antibiotic (Neosporin) (McKessonMedical-Surgical, Richmond, VA, USA). To help facilitate recovery, 1 cc of warmed sterile 0.9% saline was administered subcutaneously, and mice were kept slightly elevated on a warming pad under a thermal lamp. Following the return of normal behavior, mice were housed individually, given access to food and water *ad libitum*, and closely monitored throughout the experiment. If signs of pain or distress were observed, mice were treated with carprofen (5 mg/kg s.c.; Patterson Veterinary Supply, Inc., Loveland, CO, USA). If signs of pain or distress had continued, the veterinarian would have been consulted, and the animal would have either continued treatment or been euthanized. For this study, there was no need for a veterinarian consultation. Five days after surgery, a continuous EEG–infrared video surveillance system (Pinnacle Technology, Inc., Lawrence, KS, USA) was recorded for 48 h every 10 days. EEG recordings were acquired with a 2 kHz sampling rate and band-pass filtered between 0.5 and 40 Hz. The six KO-SD mice experienced 12 monitoring sessions, which captured 99 seizures (due to mortality, 2/6 mice had 3 sessions, 2/6 had 2 sessions, and 2/6 had 1 session). The six KO-KD mice experienced 20 monitoring sessions, which captured 68 seizures (due to increased longevity, 4/6 mice had 4 sessions, and 2/6 mice had 2 sessions).

### 2.4. Seizure Quantification

Seizures that occurred during each 48 h recording session were analyzed. All data were included in the analyses. Cortical seizures were identified via two methods. First signals were imported into Spike2 v7 software (Cambridge Electronic Design, Cambridge, UK), and time-frequency maps were generated with the fast Fourier transform. Seizures were identified based on activity in the cortical EEG and EMG traces and the time-frequency maps. Subsequently, video verification of behavioral seizures was confirmed in the original video-EEG-EMG signals in Sirenia files (Pinnacle Technology, Inc.). Severity and duration were manually determined using EEG-EMG signals and video recordings. Severity was scored using a modified Racine scale [35]: Type 1—myoclonic jerk; Type 2—head stereotypy; Type 3—bilateral clonus manifested as hunched forelimb clonus with or without rearing; Type 4—hindlimb clonus with a head tilt, tail extension with 1 or 2 rearing and falling events; Type 5—bilateral clonus and continuous rearing and falling 3 or more times; Type 6—tonic–clonic seizures involving running, energetic myoclonic jumping, falling, limb tonus and clonus. Seizure frequency, severity and duration were analyzed. To calculate a subject’s total seizure burden for each recording session, the severity and duration of each seizure were multiplied together, and the products were summed as previously described [35].

### 2.5. Sleep Architecture Quantification

Rest deficiency emerged during the last 10–15 days of life in KO-SD mice [24]. Thus, the final EEG-EMG recording prior to sudden death was used for sleep architecture quantification during the sleep phase (ZT 1:00 h–8:00 h) (Sirenia Sleep Pro; v3.0.4, Pinnacle Technologies, Lawrence, KS, USA). Recordings were divided into 10 s epochs, and sleep state was scored using a semi-automated method as we have described previously [22]. Using EEG power in the delta band of the anterior cortical electrode, located over the motor cortices and the corpus callosum, and EMG power (10–50 Hz), principal component analyses segregated epochs by state of vigilance: wake = low delta power, high EMG; NREM sleep = high delta power, low to medium EMG power; REM sleep = low delta power, low EMG. To increase rigor, EEG-EMG scoring was manually verified with a video recording. Epochs in which an animal was seizing were excluded from the sleep architecture analyses. Epochs during routine veterinary checks were excluded for all the animals. The total number of epochs analyzed averaged 2506 ± 2.4 epochs. The number of excluded epochs ranged from 3 to 39, with the fewest number of epochs excluded in the WT-SD cohort (WT-SD 3 ± 1 epochs, KO-SD 18 ± 7 epochs, WT-KD 15 ± 2 epochs, KO-KD 20 ± 3 epochs; one-way ANOVA, F (3, 17) = 4.2, *p* = 0.02). When considering the total number of epochs, less than 2% were excluded from any subject (0.54 ± 0.09% of epochs were excluded, with a range of 1.0–1.5%). EEG-EMG data were analyzed by blinded investigators. During the video verification, blinding was no longer feasible due to the spontaneous seizures of KO cohorts and the shiny coat of KD cohorts. The number of wake, NREM sleep, and REM sleep epochs was normalized to the total number of epochs analyzed within each animal.

### 2.6. Video-Noninvasive Electrocardiography (ECG)

To increase rigor, heart rate was recorded in non-anesthetized, conscious mice using a noninvasive ECG recording platform (ECGenie Mouse Specifics, Inc., Boston, MA, USA). Briefly, mice were removed from their home cage and placed on the recording platform. ECG signals were recorded for 30 min at a sampling rate of 2 kHz.

### 2.7. ECG Quantification

Traces were analyzed by blinded investigators. To determine the incidence of intermittent interictal bradycardia or tachyarrhythmia, segments of at least 25–75 P-Q-R-S-T complexes were manually reviewed to ensure signals were noise-free. Segments containing noise (i.e., in which QRS peaks could not be identified clearly and differentiated from baseline) were excluded. The average number of segments analyzed for each subject was 16.5 ± 3.5 for WT-SD mice, 14.3 ± 2.8 for WT-KD mice, 15.9 ± 1.5 for KO-SD mice, and 17.6 ± 3.8 for KO-KD mice. Mean heart rate was determined for each segment using e-MOUSE software v3 (Mouse Specifics, Inc., Boston, MA, USA). Multiple segments were analyzed per subject. Clinically, bradycardia is defined as a mean heart rate lower than the normal range of the Gaussian distribution (i.e., 60–100 bpm for humans). In this study, bradycardia was operationally defined as a mean heart rate that was less than twice the standard deviation of the WT Gaussian distribution, as described previously [4].

### 2.8. Video-Noninvasive Airway Mechanics

Following the ECG recording, mice were acclimated to a noninvasive airway mechanics chamber (NAM; Buxco FinePointe/DSI Inc., Minneapolis, MN, USA) for 10–15 min, followed by 3 min of data collection. Data were acquired with Buxco FinePointe acquisition software V2.3.1.9. There were 45 respiratory monitoring sessions for 7 WT-SD mice, 39 sessions for 8 WT-KD mice, 39 sessions for 10 KO-SD mice, and 39 sessions for KO-KD mice.

### 2.9. Apnea Quantification

Traces were analyzed by blinded investigators and manually inspected. Segments containing noise (i.e., a seizure) were excluded from analyses. Traces were manually reviewed to quantify apnea. In humans, respiratory rates range from 12 to 18 breaths per min., and apnea is defined as the cessation of breathing for 10 s, which equates to 2–3 respiration cycles. To ensure assay sensitivity herein, apnea was defined as a decrease in airflow ≥90% for a period of two or more complete respiratory cycles. The respiratory cycle was calculated for each subject at baseline using the following equation: 2 * (frequency of breaths min^−1^/60 s).

### 2.10. Video-Oximetry

To increase rigor and minimize potential stress effects of other experiments, a separate cohort was used to measure arterial blood oxygen saturation (SaO_2_) noninvasively using video-synchronized MouseOx Plus infrared pulse oximetry (Starr Life Sciences Corp, Oakmont, PA, USA), as we have described previously [4]. Following removal of the hair around the neck, the mice were weighed, and an appropriately sized CollarClip Sensor (Starr Life Sciences Corp) was placed around the neck of the animal. Eight parameters, including SaO_2_, pulse rate and breathing rate, were acquired at 5 Hz, and mice were recorded for 1 h. Behavior (rest, active, and seizure) was manually recorded during the session. Each time a behavior changed, the time was noted. Behaviors were also verified manually with video *post hoc*.

### 2.11. SaO_2_ Quantification

Signal fidelity was considered reliable when all eight parameters were successfully measured (i.e., designated error-free). If a measurement successfully detected SaO_2_ but failed on any other parameter, it was excluded. Only completely error-free measurements were included in the analyses. The time-stamped behavior (rest, active, and seizure) was identified for each measurement. If a recording session had fewer than 50 error-free measurements throughout, the entire recording session was excluded. The number of monitoring sessions that resulted in a sufficient number of error-free measurements sampled throughout the recording were 22/22 for WT (*n* = 12 mice), 8/9 for KO-SD (*n* = 5 mice; two mice had only one recording session before sudden death, and three mice had two monitoring sessions before death), and 21/25 for KO-KD (*n* = 8 mice). During each recording session, the average number of error-free measurements per subject was 1530 ± 500 for WT mice, 507 ± 98 for KO-SD, and 522 ± 144 for KO-KD. Data points below 90% SaO_2_ were demarcated as hypoxemia.

### 2.12. Statistical Analyses

Data are presented as the mean ± standard error of the mean. Statistical significance was determined with Prism10 software (Graphpad Software Inc., La Jolla, CA, USA). Survival curves of cohorts were compared using the log-rank Mantel–Cox test. Normality assumptions were tested before applying parametric tests. Two-way ANOVA, effect size (η^2^), and Šidák’s multiple comparison *post hoc* test were used to analyze prospective seizure data and retrospective seizure, sleep architecture, heart rate, apnea, and the hypoxia fraction. The chi-square test assessed apnea susceptibility. Oximetry data did not meet normality assumptions; thus, the Kruskal–Wallis test with Dunn’s multiple comparisons *post hoc* test was used.

## 3. Results

### 3.1. KD Treatment Increases Lifespan of KO Mice

**Figure 2:**
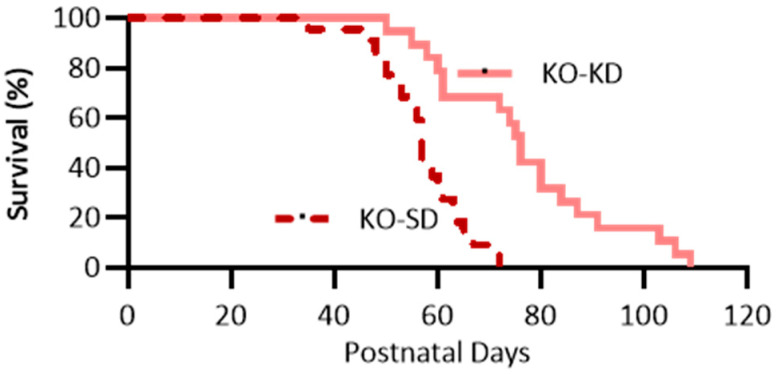
Effect of ketogenic diet on longevity. Kaplan–Meier survival curves of KO-SD (red hatched) and KO-KD (salmon) cohorts. KD treatment significantly delayed the mean age of sudden death from P57 ± 2 to 77 ± 4 days, *n* = 22 KO-SD and 19 KO-KD, *p* < 0.001.

To verify the reproducibility of [18], we tested the hypothesis that KD treatment would increase longevity. The Kaplan–Meier analysis was used to compare the survival curves of the KO-SD and KO-KD groups (Figure 2, cohorts from Experiments I and II). KD treatment significantly delayed the mean age of sudden death from P57 ± 2 to 77 ± 4 days (*n* = 22 SD and 19 KD, *p* < 0.001). Overall, comparison of hazard ratios of the two groups indicates that KO-SD mice were 3.2 times more likely to die younger compared to the KO-KD group (hazard ratio = 3.2, CI: 1.6–6.4). While KO-KD mice still experienced premature mortality, treatment did increase longevity, corroborating previous findings.

### 3.2. Experiment I

Experiment I verified the reproducibility that KD would exert antiseizure and somnogenic effects. In Experiment I, previous studies were further elaborated by employing EEG-EMG for REM sleep and NREM sleep quantification and analyzing seizure and sleep data relative to the day of sudden death (described in detail below).

#### 3.2.1. KD Treatment Reduced the Number of Seizures Experienced by KO Mice During the Last 10 Days of Life

**Figure 3:**
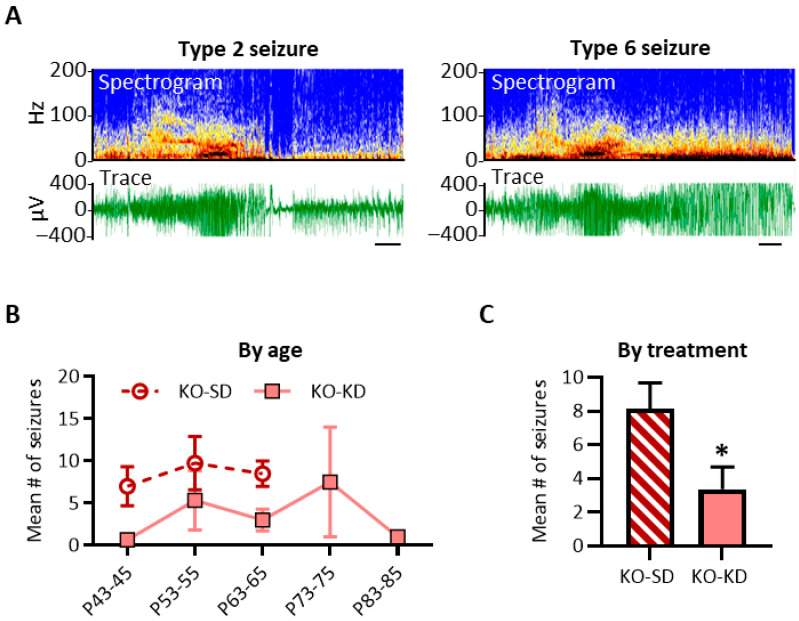
Prospective effect of ketogenic diet on seizures in KO mice. (**A**) Representational examples of EEG traces and respective spectrograms of Type 2 and Type 6 generalized seizures from KO-SD mice. The blue-red spectrum provides a visual depiction of the amount of power each frequency contributes to the signal (blue = no contribution; deep red = strongest contribution). Trace amplitude surpassed the −400–400 µV acquisition range. Horizontal scale bar = 5 s. (**B**) Number of seizures temporally plotted prospectively by age during each 48 h recording session (due to mortality, *n* for P43–45: 6 KO-SD, 6 KO-KD mice; P53–55: 4 KO-SD, 6 KO-KD mice; P63–65: 2 KO-SD, 4 KO-KD mice; P73–75: 2 KO-KD mice; and P83–85: 1 KO-KD mouse). (**C**) Mean number of seizures experienced by each subject across all recording sessions *(n* = 6 mice per group). Values represent the mean ± SEM, * *p* < 0.05.

To verify the reproducibility of KD-induced seizure reduction [18,22,30], we tested the hypothesis that KD treatment would exert antiseizure effects when compared with age-matched KO-SD mice. This experiment expands upon previous studies in two ways: First, EEG was utilized in addition to behavior to identify and score seizures. Example traces of Type 2 and Type 6 seizures with EEG and fast Fourier time-frequency signatures are depicted in Figure 3A. KD exerted a significant antiseizure effect when data were prospectively aligned by age (diet effect: F (1, 22) = 5.663, *p* < 0.05; η^2^ = 0.19; however, there were no age-specific *post hoc* differences) (Figure 3B) and when collapsed across ages (*p* < 0.05, unpaired two-tailed *t*-test with Welsh’s correction) (Figure 3C), corroborating previous findings.

**Figure 4:**
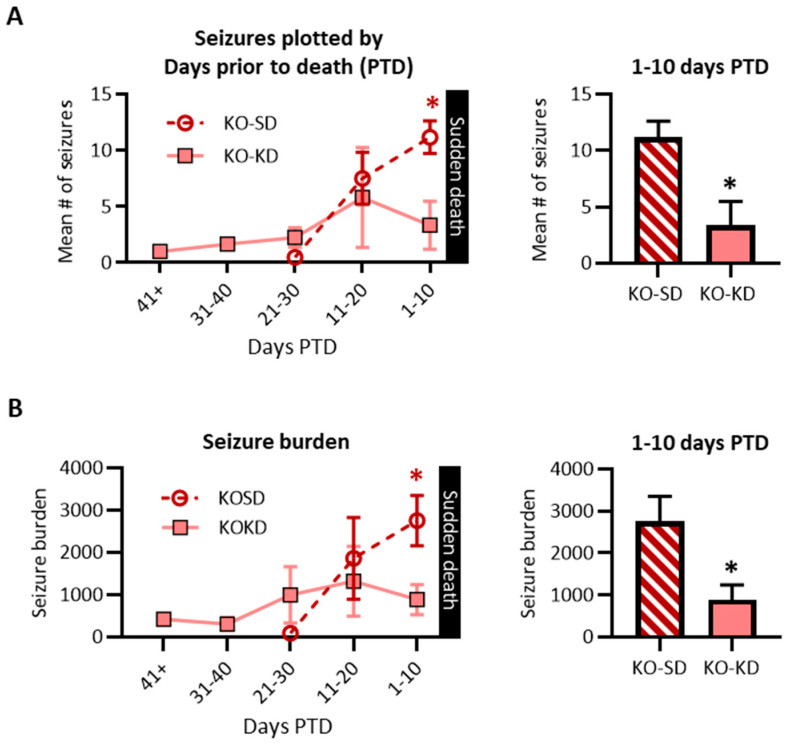
Retrospective effect of ketogenic diet on KO seizures. (**A**) Seizure number plotted retrospectively by the number of days prior to death (PTD) (*n* for PTD41+: 1 KO-KD mouse; PTD31–40: 3 KO-KD mice; PTD21–30: 2 KO-SD, 4 KO-KD mice; PTD11–20: 4 KO-SD, 5 KO-KD mice; PTD1–10: 6 KO-SD, 6 KO-KD mice). The bar graph depicts seizure numbers 1–10 days PTD. (**B**) Seizure burden plotted retrospectively by the number of days PTD. The bar graph depicts seizure burden 1–10 days PTD (*n* = 6 mice per group). Values represent the mean ± SEM, * *p* < 0.05.

The second novel aspect of this experiment is that the analysis controls for (i) the extended longevity of KD-treated cohorts and (ii) the wide age range of mortality. Data were realigned by the day of sudden death and replotted in days ‘prior to death’ (PTD). Using this retrospective analysis, we tested the hypothesis that KD treatment would continue to exert antiseizure effects during the last two weeks of life. With the EEG monitoring occurring every 10 days, if a subject experienced sudden death the day after their third session, data from the last session would be plotted at 1 day PTD, the previous session would be plotted at 11 days PTD, and the first session would be plotted at 21 days PTD. Similarly, if a subject experienced death 5 days after their fourth session, their last session would be plotted at 5 days PTD, the previous session would be plotted at 15 days PTD, the second session would be plotted at 25 days PTD, and the first session would be plotted at 35 days PTD. Due to this variability, data for each cohort were pooled into 10-day bins to ensure sufficient power (i.e., 1–10 days PTD, 11–20 days PTD, etc.) (Figure 4A).

KD-treated mice regularly experienced 0–4 seizures during 16/19 monitoring sessions. Overall, KO-KD mice had significantly fewer seizures 1–10 days PTD (F (1, 21) = 1.2, *p* < 0.3; Šídák’s *post hoc* test 1–10 days PTD, *p* < 0.05; PTD effect size η^2^ = 0.14) (Figure 4A). Seizure severity or duration did not differ between treatment cohorts at each PTD (severity: F (2, 155) = 2.87, *p* = 0.06; and duration: F (2, 155) = 0.09, *p* = 0.91); thus, overall, the seizure burden of each subject during each recording session was determined. Seizure burden was significantly reduced in KO-KD mice 1–10 days PTD (Šídák’s *post hoc* test, *p* < 0.05). Data indicate that KD treatment attenuated seizures in KO mice during the last 10 days of life.

#### 3.2.2. KD Treatment Does Not Restore the Abnormal Sleep Architecture of KO Mice During the Last 10 Days of Life

Rest–activity cycles of KO-SD mice generally become deficient in the last 10–15 days of life, and KD treatment failed to normalize the deficiency [24,30]. Here, we verified reproducibility and further expanded upon these studies with EEG-EMG monitoring to test the hypotheses that KO-SD mice were deficient in both NREM sleep and REM sleep and that KD treatment would not restore the sleep architecture deficits in the last 10 days of life.

**Figure 5:**
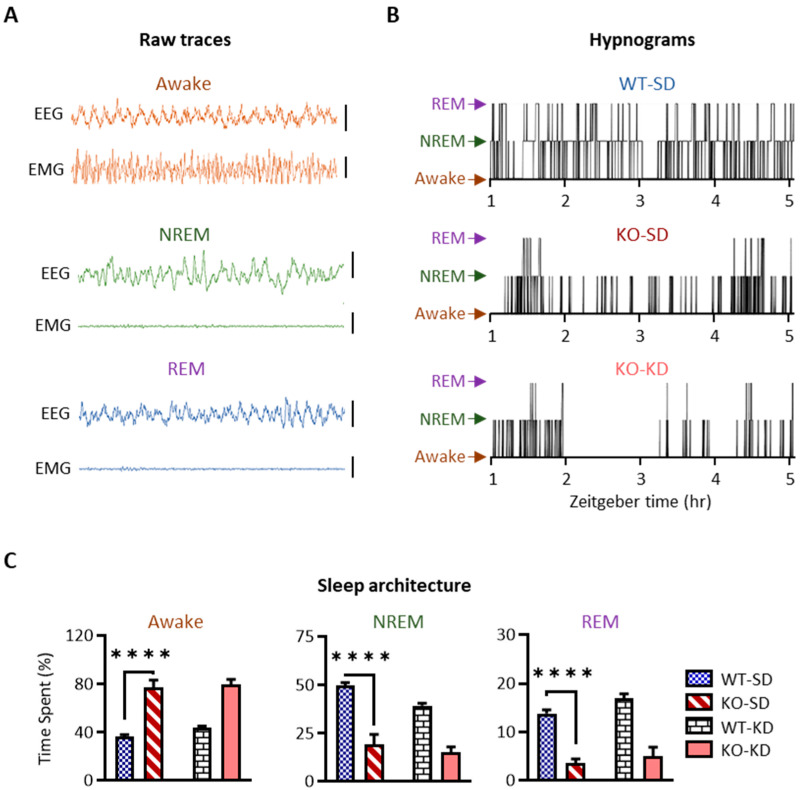
Effect of ketogenic diet on sleep architecture 1–10 days PTD. (**A**) Representative EEG and EMG traces during awake (orange), NREM sleep (green) and REM sleep (purple) epochs. Each trace is 5 s. Vertical EEG scale bars are 0.4 mV, and EMG scale bars are 80 µV. (**B**) Representative hypnograms from WT-SD, KO-SD, and KO-KD mice during a 4 h period provide temporal depictions of each transition (vertical line) between awake (bottom row of each hypnogram), NREM sleep (middle row of each hypnogram) and REM sleep (top row of each hypnogram). (**C**) KO-SD mice spent more time awake and less time in NREM sleep and REM sleep. KD treatment had no impact. WT-SD, blue hatched bars; KO-SD, red diagonal striped bars; WT-KD, white brick bars; KO-KD, salmon bars; *n* = 5–6 mice in each treatment group and genotype, **** *p* < 0.0001.

Representative EEG and EMG traces during wake, NREM sleep, and REM sleep are depicted in Figure 5A. Sleep architecture was disrupted in KO-SD mice, with more epochs spent awake (F (1, 17) = 104.10, *p* < 0.0001) and less in NREM sleep and REM sleep (NREM: F (1, 17) = 85.67, *p* < 0.0001; REM: F (1, 17) = 95.16, *p* < 0.0001) when compared to age-matched WT-SD controls (hypnograms in Figure 5B and quantification in Figure 5C) (genotype η^2^ = 0.83, 0.75, 0.83). With minimal impact on WT NREM sleep (F (1, 17) = 6.08, *p* = 0.03; Šidák’s *post hoc* analyses WT-SD v WT-KD, *p* < 0.02), KD treatment did not restore NREM sleep in KO-KD mice (*p* = 0.35). In addition, treatment with KD did not impact REM sleep (F (1, 17) = 4.28, *p* = 0.054; Šidák’s *post hoc* analyses KO-SD v KO-KD, *p* = 0.59) or wake (F (1, 17) = 1.70, *p* = 0.21) of KO mice 1–10 days PTD (Figure 5C). These findings indicate that KD treatment did not restore sleep deficiency during the last 10 days of life.

### 3.3. Experiment II

Experiment II tested the hypotheses that (i) bradyarrhythmia and apnea would increase in the days prior to death and (ii) KD treatment would determine whether the emergent bradycardia and/or apnea persisted as temporal biomarkers in preclinical SUDEP.

#### 3.3.1. KD Treatment Attenuated the Emergence of Intermittent Interictal Bradycardia in KO Mice During the Last 20 Days of Life

**Figure 6:**
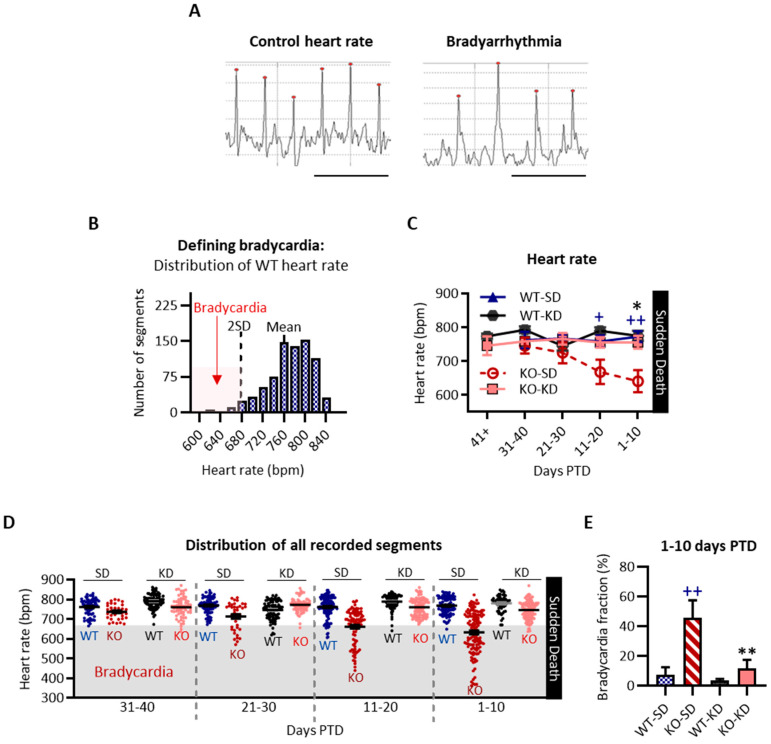
Retrospective effect of ketogenic diet on heart rate. (**A**) Representative electrocardiography (ECG) traces depicting a normal rhythm and bradycardia. The red dot indicates ‘R’ in the QRS complex. Horizontal scale bar 0.2 s. (**B**) A histogram of the distribution of WT heart rate data. The mean and 2 standard deviations (2 SD) below the mean are indicated. Bradycardia was identified as a heart rate less than 2 SD, or 684 bpm. WT-SD and WT-KD heart rates did not differ (*p* > 0.9 between groups, *p* > 0.9 at each timepoint) and were thus combined to determine bradycardia. (**C**) Heart rate data plotted retrospectively in days PTD. (**D**) Scatterplot of the mean heart rate for each segment from WT-SD (blue), KO-SD (red), WT-KD (black), and KO-KD (salmon) recordings. Segments with a mean rate of <684 are considered bradycardia and are highlighted in the shaded region. (**E**) The bradycardia fraction was determined for each subject (number of segments identified as bradycardia/total number of segments). Sample sizes = 6–7 mice for each genotype and each diet treatment. All values represent mean ± SEM. ^+^ *p* < 0.05, ^++^ *p* < 0.01 when comparing KO-SD vs. WT-SD. * *p* < 0.05, ** *p* < 0.01 when comparing KO-SD vs. KO-KD.

Clinical bradycardia is defined as a heart rate below the normal range (i.e., <60 bpm). Example traces of normal heart rate and bradycardia are provided in Figure 6A. First, the control range of the WT heart rate was determined. The mean WT heart rate was 770 ± 43 bpm (mean ± SD), with a range of 607–862 bpm and 95% of values within ±2 SD of the mean (Figure 6B). Accordingly, an ECG segment was classified as bradycardia if the rate was 2 SD lower than the WT mean (i.e., less than 684 bpm) (Figure 6B).

Heart rate for all cohorts was plotted in days PTD as a line graph (Figure 6C). At 21–30 days, the KO-SD heart rate (725 ± 31 bpm) resembled age-matched WT-SD (767 ± 11 bpm, *p* = 0.42), WT-KD (745 ± 14 bpm, *p* = 0.89) and KO-KD cohorts (766 ± 16 bpm, *p* = 0.60) (Figure 6C). This suggests normal cardiac function in KO-SD mice 21–30 days PTD.

Intermittent interictal bradyarrhythmia emerged in KO-SD mice thereafter (F (1, 30) = 17.00, *p* < 0.001; 11–20 PTD: KO-SD 668 ± 35 bpm vs. WT-SD 758 ± 17 bpm, *p* < 0.05; 1–10 days PTD: KO-SD 641 ± 32 bpm vs. WT-SD 772 ± 15 bpm, *p* < 0.01). KD treatment did not alter WT-SD heart rates (F (1, 31) = 0.1069, *p* = 0.745) but did mitigate the emergent bradyarrhythmias in KO-SD mice 1–10 days PTD (F (1, 26) = 11.58, *p* < 0.01, treatment η^2^ = 0.15).

A scatterplot of all segments analyzed depicts the proportion of segments that were defined as bradycardia (Figure 6D). The bradycardic fraction was determined for each animal (bradycardia segments/total segments). KO-SD mice had a greater bradycardic fraction compared to WT-SD controls (F (1, 24) = 10.74, *p* = 0.003; genotype effect size η^2^ = 0.23; KO-SD 45.7 ± 11.8% vs. WT-SD 7.2 ± 5.2%, respectively, *p* < 0.01) (Figure 6E). KD treatment markedly reduced the bradycardic fraction in KO mice (F (1, 24) = 7.15, *p* = 0.01; treatment effect size η^2^ = 0.15), which resembled WT-SD mice (*p* = 0.99). These findings indicate that KD treatment attenuated the emergence of interictal bradycardia in KO mice during the last 20 days of life.

#### 3.3.2. KD Treatment Attenuated the Emergence of Apnea in KO Mice During the Last 10 Days of Life

The hypotheses that (i) apnea would increase in the days prior to death and (ii) KD treatment would determine whether emergent apnea persisted as temporal biomarkers in preclinical SUDEP were tested.

**Figure 7:**
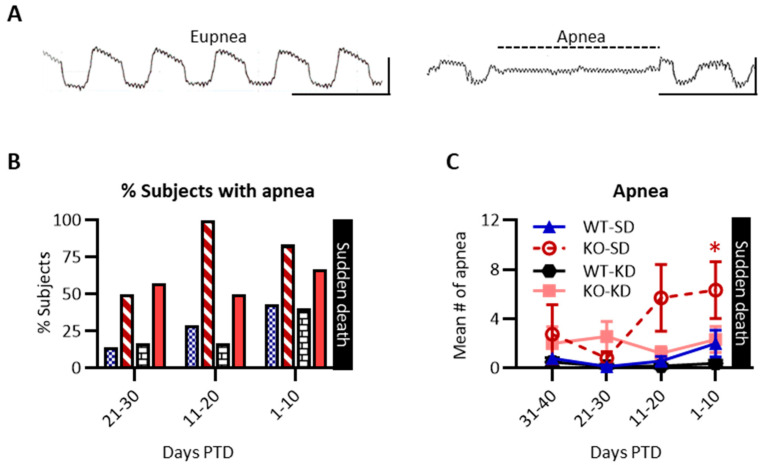
Retrospective effect of ketogenic diet on apnea. (**A**) Example traces of eupnea (normal breathing rate) on the left and apnea on the right. Horizontal scale bar: 0.5 s and vertical scale bar: 6 mL. (**B**) Bar graph depicting the percentage of subjects that experienced apnea per cohort at each timepoint: WT-SD (blue speckled, *n* = 7), KO-SD (red striped, due to mortality, *n* = 12 at 1–10 PTD, 7 at 11–20 PTD, 4 at 21–30), WT-KD (black hatched, *n* = 6), and KO-KD (salmon, *n* = 8–10). Due to early mortality for the KO cohorts, there are fewer subjects at P21–30 days PTD. Note: χ^2^ statistics reported in the Results section combined WT-SD and WT-KD data to increase power. (**C**) Line graph depicting the mean number of apneic episodes for WT-SD (blue triangle), KO-SD (red empty circle), WT-KD (black hexagon), and KO-KD (salmon square) cohorts, * *p* < 0.05.

Apnea is defined as the cessation of breathing for two respiratory cycles (Figure 7A). KD did not alter apnea susceptibility or incidence in WT controls, indicating that KD does not promote apnea in otherwise healthy animals (*p* = 0.98); thus, data were combined to determine susceptibility: The proportion of KO-SD mice susceptible to apnea increased during the last 20 days of life when compared to WT cohorts (11–20 days PTD (*χ*^2^ (1, 23) = 13.08, *p* < 0.001); 1–10 days PTD (*χ*^2^ (1, 25) = 4.44, *p* < 0.05)) (Figure 7B).

The mean number of apneic events experienced 20+ days PTD was similar among groups (i.e., 21–30 PTD: KO-SD 0.8 ± 0.5; KO-KD 2.6 ± 1.2; WT-SD 0.1 ± 0.2; WT-KD 0.2 ± 0.2 apneic episodes). However, during the last 10 days PTD, KO-SD mice experienced a higher incidence of apnea (F (1, 91) = 4.22, *p* = 0.008; η^2^ = 0.10) when compared with earlier timepoints (i.e., 21–30 days PTD 0.8 ± 0.5 episodes vs. 1–10 days PTD 6.3 ± 2.3 episodes, *p* < 0.05) (Figure 7C). In contrast, the low number of apneic episodes for the other cohorts did not differ across timepoints (*p* > 0.5). These data suggest KD treatment attenuated the emergence of apnea in KO mice during the last 10 days of life.

### 3.4. Experiment III

Experiment III tested the hypotheses that (i) intermittent hypoxemia would increase in the days prior to death and (ii) KD treatment would determine whether the hypoxemia persisted as temporal biomarkers in preclinical SUDEP.

#### KD Partially Attenuated Intermittent Interictal Hypoxemia in KO Mice During the Last 20 Days of Life

**Figure 8:**
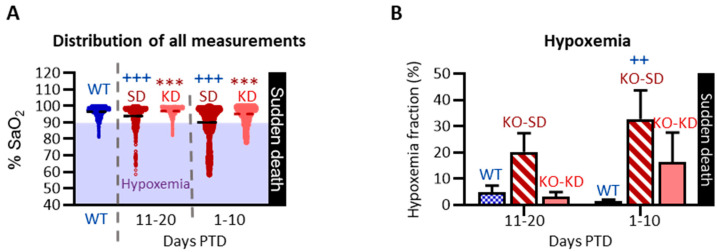
Retrospective effect of ketogenic diet on blood oxygen saturation. (**A**) Scatterplot of all raw SaO_2_ values recorded from WT (blue, combines both WT-SD and WT-KD), KO-SD (red) and KD-KD (salmon) cohorts. Recordings of <90% SaO_2_ are considered hypoxemic (highlighted in the shaded region). Blood oxygen desaturation (<90% SaO_2_) is more apparent in KO-SD mice during the last 20 days of life. KD treatment significantly reduced the incidence of intermittent interictal hypoxemia (15,989 measurements total from 6 KO-SD, 8 KO-SD and 12 WT mice). (**B**) The hypoxemia fraction was determined for each subject (the number of hypoxemia measurements/total number of error-free measurements for each subject). All values represent mean ± SEM. *** *p* < 0.001 when comparing PTD-matched KO-SD and KO-KD cohorts; ^++^ *p* < 0.01 and ^+++^ *p* < 0.001 when comparing WT and KO-SD cohorts.

SaO_2_ measurements from WT-SD and WT-KD did not differ (*p* > 0.05); thus, data were pooled (Figure 8). There were no differences in SaO_2_ measurements among behaviors (*p* > 0.05), so data were combined. As depicted in the scatterplot, KO-SD mice had significantly more SaO_2_ recordings that dropped below 90%, indicating increased intermittent hypoxemia (H(4) = 1463, *p* < 0.001) (Figure 8A). KO-SD mice had a lower mean SaO_2_ 11–20 days PTD compared to WT controls (KO-SD 93.92 ± 0.13% vs. WT 96.48 ± 0.04%, respectively, *p* < 0.001). The mean SaO_2_ of KO-SD mice further reduced to 90.05 ± 0.23% 1–10 days PTD, differing significantly from ten days earlier (*p* < 0.001) and when compared to both WT controls (*p* < 0.001) (Figure 8A). KD-treated KO mice had stable SaO_2_ levels when compared to KO-SD cohorts (1–10 days PTD 95.13 ± 0.08% and 11–20 days PTD 96.83 ± 0.06%, respectively, *p* < 0.001). Hypoxemia fraction for each subject indicates KO-SD mice 1–10 days PTD had ~33% of their SaO_2_ measurements registered as hypoxemic (F (2, 32) = 5.65, *p* < 0.01; genotype effect η^2^ = 0.23; diet effect η^2^ = 0.04) (Figure 8B). The hypoxemic fraction of KD-treated KO mice did not differ from KO-SD or WT cohorts. Data suggest that KD treatment attenuated the overall total number of hypoxemic segments and trended to attenuate the hypoxemia fraction during the last 10 days of life.

## 4. Discussion

There are four novel findings in this study. To our knowledge, this is the first study to systematically and comprehensively evaluate the longitudinal effects of KD treatment in preclinical SUDEP. Replicating previous data, KD treatment significantly increased longevity and reduced seizures. Using retrospective analyses from the day of death, KD treatment attenuated the emergence of (i) interictal intermittent bradycardia in the last 20 days of life, (ii) apnea, and (iii) intermittent hypoxemia in the last 10 days of life. In contrast, (iv) KD treatment did not rescue REM and NREM sleep deficiencies during the last 10 days of life.

### 4.1. KD Seizures and Survival

KD is an effective and safe treatment for refractory epilepsy with evidence of minimal or manageable side effects [27,28,36]. KD significantly extended the lifespan of Kv1.1 KO mice by ~35%, supporting our previous findings and studies in the *SCN1a* model of Dravet Syndrome [18,24,37,38]. These converging results across genetic SUDEP models highlight the longevity-promoting effects of KD in epilepsy. While a reduction in severe seizures may contribute to extended longevity, it is insufficient to prevent premature mortality herein. This supports clinical observations that individuals either with well-controlled seizures or who have been seizure-free for a year still succumb to SUDEP [10]. These findings further support the current notion that while seizure severity and frequency are critical risk factors, SUDEP is multifactorial.

### 4.2. KD and Heart Rate

Postictal bradycardia is a SUDEP risk factor in clinical cohorts and in preclinical rodent and rabbit SUDEP models [4,8,16,20,21,39,40]. The few reports that have characterized the impact of KD on heart function were focused on overall cardiac structure, contractility and monitoring potential adverse effects rather than intentional therapeutic outcomes [41,42,43]. Long-term monitoring of children with drug-resistant epilepsy indicates that the KD does not adversely affect ventricular systolic or diastolic function or cardiac structure [41,42,43]. The rare cases of KD-induced QT prolongation and sudden cardiac death are often concurrent with selenium deficiency [44]. Additional studies report that KD therapy improves cardiac muscle metabolism and energy dynamics, regulates cardiac vagal and parasympathetic tone, and influences cholesterol pathways, all of which can impact heart rate and bradyarrhythmia [45,46,47,48]. Whether these mechanisms contribute to the KD’s attenuation of emergent intermittent bradycardia herein requires further study. Outside KD’s direct impact on the heart, cardiac function is interdependent with respiratory function and blood gas stability; thus, effects herein may be indirect.

### 4.3. KD and Apnea

Blood gas instability occurs in approximately thirty percent of generalized convulsive seizures and consequent apneas in the form of severe peri-ictal hypoxemia and hypercapnia, which persist anywhere from 60 s to 30 min [4,9,19,49,50,51,52]. To our knowledge, there are no clinical or preclinical reports evaluating the impact of KD treatment on central apnea in epilepsy. However, KD treatment reduces sleep apnea in people with relapsing multiple sclerosis [53] and in people with obesity hypoventilation syndrome [31]. Data herein support these findings and suggest the ability of KD to reduce apnea extends to preclinical epilepsy. An interesting, underpowered observation noted that four KO-KD mice did not experience apnea, whereas all KO-SD mice experienced apnea at least once across multiple recording sessions. This possible protective effect against apnea onset requires further study.

### 4.4. Metabolic Therapies and Blood Gas Stability

There are limited reports evaluating the impact of KD treatment on blood oxygen saturation or blood gas stability [31,32,54]. In people with obesity hypoventilation syndrome, two weeks of KD treatment improves nocturnal blood oxygenation saturation and reduces CO_2_ accumulation (venous CO_2_) by 3 mm Hg [31]. The recognized beneficial effects of KD on these and other respiratory endpoints (including reducing respiratory quotient [31] and the respiratory exchange ratio in healthy individuals [32]) have led to the proposed use of KD as an adjuvant respiratory therapy in obese individuals with COVID-19 [55]. Treatment with a low-caloric therapy (which also promotes a ketogenic state) attenuates the severity and frequency of hypoxemia in people with chronic hypercapnic respiratory failure [56]. In a preclinical seizure model, KD attenuates postictal hypoxia and improves brain oxygen levels (80). These data support the KD-mediated reduction in the overall number of hypoxemic measurements. Herein, the overall SaO_2_ was improved, and the hypoxemia fraction of KO-KD mice did not differ from KO-SD 1–10 days PTD or WT controls; thus, data should be cautiously interpreted.

### 4.5. Central Chemosensitivity and Epilepsy

Our previous studies demonstrated that KO-SD mice at high risk of SUDEP experience chronic intermittent oxygen desaturation during seizures and apneas–hypopneas but also during normal behavior [4,19]. Atypical, intermittent or prolonged blood gas instability (hypercapnic and hypoxemic) may result from dysregulated chemosensitivity in central regions. Indeed, functional fMRI of conscious subjects found hypercapnic challenges activate atypical central circuitry in PWE when compared to the chemoresponsive circuitry activated in controls [57]. This supports the notion that central chemoresponsive circuitry is different in PWE. While some chemoresponsive regions have been identified in epilepsy and SUDEP models (i.e., orexin neurons in the lateral hypothalamus, neurons in the retrotrapezoid nucleus, and serotonin neurons in the raphe nucleus) [4,58,59,60,61,62], the extent of changes throughout the circuitry requires further study.

One vital factor to surviving apnea is the body’s ability to trigger breathing (the autoresuscitation response), which depends on functional central chemosensitivity. The landmark MORTEMUS study provided critical insights into the terminal sequence of SUDEP: a severe seizure followed by transient bradyarrhythmias and apneas, then terminal apnea from which the individual does not autoresuscitate, and subsequent asystole [8]. Central chemosensitive regions that respond to hypercapnia and hypoxia typically trigger the autoresuscitation response from long-duration apnea [59,63,64,65]. Preclinical studies support the notion that the autoresuscitation response is dysfunctional in high-SUDEP-risk cohorts and fails to trigger survival at sudden death [63,66], the extent of the circuitry dysfunction is unclear. The impact of KD on chemoresponsive regions is unknown.

However, despite KD prolonging lifespan herein, the autoresuscitation circuitry remained dysfunctional in KO-KD cohorts, as it failed to promote survival, and mice succumbed to sudden death. Similarly, KD treatment was able to prolong the latency to seizure-induced respiratory arrest in preclinical Dravet syndrome models; however, here again, the mice ultimately failed to autoresuscitate [37,38].

### 4.6. KD and Sleep

Herein, during the last 10 days of life, KD treatment was also unable to prevent the deficiencies in REM sleep and NREM sleep in KO-KD mice. Lack of the Kv1.1 protein is unlikely to contribute to sleep deficiency, as it cannot account for the emergent property. Speculative factors that may contribute to sleep deficiency include neurodegeneration, cumulative seizure burden, and chronic physiological stress. Sleep deficiency is associated with increased levels of central orexin [4,22]. Importantly, blockade of orexin receptors increases NREM sleep and reduces time spent awake [4,22], indicating the deficiency is acutely reversible, irrespective of the precipitating cause.

The KD has complex effects on sleep that are not fully understood [23,24,29,53,67,68,69]. For example, six months of KD treatment improves insomnia in people with relapsing multiple sclerosis [53]. Despite KD treatment reducing Stage 2 NREM sleep and total nighttime sleep in children with drug-resistant epilepsy, REM sleep is increased [29,67]. Other reports indicate that KD qualitatively improves sleep in PWE [68,69]. As each sleep state is a manifestation of specific neurobiological–neurophysiological circuitry, perhaps KD exerts unique impacts on NREM sleep and REM sleep promoting regions in a disease-specific manner [70]. Actimetry data indicate KD treatment rescues the rest deficiency in KO-KD mice up until the last two weeks of life [24]. Similarly, during the last 10 days of life, KD treatment did not prevent the reductions in both REM sleep and NREM sleep in KO mice.

Reduced REM sleep is linked to increased risk of heart failure, arrhythmias, hypertension, and mortality [71,72,73]. Preclinically, sleep deprivation promotes hypoxemia, reduced baroreflex sensitivity, and altered brainstem signaling, all of which can promote blood gas instability [74,75,76,77]. Clinically, chronic sleep deficiency exacerbates seizures, impairs autonomic regulation, and alters ventilatory responses to hypoxemia [78,79,80,81,82,83]. Future studies are required to determine whether resolving sleep deficiency is sufficient to reduce SUDEP risk.

The simplest explanation for early mortality herein is the lack of the Kv1.1 protein. However, lack of Kv1.1 protein in other murine background strains did not result in 100% mortality [15], suggesting it is not the cause but may contribute to susceptibility. The Kv1.1 knockout mouse model on the C3HeB/FeJ background strain is useful for SUDEP because mortality can be delayed, which in itself holds clinical relevance. However, whether additional unidentified factors synergistically promote sudden death and/or whether another therapeutic approach can prevent sudden death in this model is the topic of current and future investigation.

### 4.7. Biomarkers and SUDEP

While seizures, bradyarrhythmia and apnea were considered temporal biomarkers in KO-SD cohorts, they did not persist in KO-KD cohorts. Biomarkers investigated herein may be part of a broader pathophysiology, including brainstem pathology or autonomic dysregulation that synergistically drives mortality through other parallel pathways despite modification of these biomarkers. For example, while bradycardia and heart rate are stabilized in KO mice, if an underlying autonomic dysfunction is not stabilized by KD, it could still precipitate atrioventricular blocks that result in cardiac asystole and death [84]. Thus, while KD-induced modulation of these biomarkers is therapeutically still valuable, their predictive power in the context of mortality and SUDEP needs further evaluation.

### 4.8. Limitations

This study has several limitations. Lack of concurrent EEG–ECG–respiration recordings constrains interpretation. Cardiac output, atrioventricular (AV) block and other measures of cardiac function that may contribute to cardiac death independent of bradyarrhythmias were not measured. Due to the invasive nature of the EEG-EMG recordings, continuous monitoring was not chosen; thus, the sampling strategy did not capture lifetime cumulative seizure burden or sleep deficiency. No subject experienced sudden death during a recording, so it is unclear whether bradyarrhythmia and apnea preceded terminal apnea. Within each cohort, there were 3–4 males and females; while sex differences were not observed, the study did not have sufficient power for statistical determination. While alignment of data with proximity to death provided insights, it remains unclear whether changes are indicative of terminal decline, cumulative disease burden, or true predictive biomarkers. Future studies will address these limitations.

## 5. Conclusions

Our findings provide novel preclinical support for KD as a candidate therapy to attenuate seizure frequency and burden, bradycardia, apnea, and hypoxemia in SUDEP. Sleep deficiency persists as a potential temporal biomarker of preclinical SUDEP; however, causality was not tested herein. Determining whether it contributes mechanistically to SUDEP is a critical need for future investigations. Speculating on the generalizability to PWE, these findings support the current notion that while seizure severity and frequency are critical risk factors, SUDEP is multifactorial. To better understand the impact on mortality with respect to patient heterogeneity, data herein strengthen the rationale for prospective clinical trials to include real-time monitoring of heart rate, apnea, blood oxygen saturation, and at-home sleep quality (vs. in-clinic sleep, which is often interrupted) with wearable devices in high-risk PWE.

## Data Availability

The original contributions presented in this study are included in the article. Further inquiries can be directed to the corresponding author.
